# Brain neural mechanisms underlying VR enhanced aerobic exercise for mood enhancement in depressed adolescents

**DOI:** 10.3389/fnhum.2025.1706793

**Published:** 2026-01-12

**Authors:** Shuqi Yao, Guochen Wang, Ting Peng, Longhai Zhang, Fuhai Ma, Puyan Chi

**Affiliations:** 1 School of Physical Education, Shaanxi Normal University, Xi’an, China; 2 SMS Longhua School, Shenzhen, China; 3 Department of Physical Education, Shanghai Maritime University, Shanghai, China; 4 School of Physical Education, Qinghai Nationalities University, Xining, Qinghai, China

**Keywords:** adolescents, aerobic exercise, depressive symptoms, electroencephalography, virtual reality

## Abstract

**Introduction:**

To compare the immediate effects of a single bout of virtual reality (VR) aerobic exercise versus traditional aerobic exercise on depressive mood in middle-school students and to explore the underlying Electroencephalogram (EEG) microstate and power-spectrum mechanisms.

**Methods:**

Forty middle-school students were classified into depressed and healthy groups based on the PHQ-9 and completed 15 min of moderate-intensity conventional cycling and VR cycling in a crossover design. Mood was assessed with the Brief Mood Scale (BFS) before and after each intervention, and resting-state EEG was recorded. EEG signals were processed using power spectrum analysis and microstate analysis, and correlation analysis was conducted between BFS questionnaire scores and microstate parameters.

**Results:**

Both interventions significantly increased vigor and pleasure while reducing depression and lethargy (*p* < 0.001); VR was superior to traditional cycling in improving vigor, depression, and lethargy (*p* < 0.01). At baseline, the depressed group showed elevated occurrence and contribution of microstate C with abnormal centroids. After exercise, microstate distributions normalized in both groups; VR specifically reduced microstate C occurrence (*p* < 0.05), prolonged microstate D duration (*p* < 0.05), and enhanced B → C and D → B transitions (*p* < 0.05). Post-VR B → C transition rate correlated negatively with depression (*r* = −0.462), whereas microstate D occurrence correlated positively with pleasure (*r* = 0.450). Relative theta, alpha, and beta power decreased after exercise (*p* < 0.01), without additional VR-mediated power-spectrum gains.

**Conclusion:**

A single bout of VR-assisted aerobic exercise more effectively enhances immediate mood in middle-school students with depressive symptoms than traditional cycling, likely owing to stronger modulation of microstate dynamics linked to emotion and attention networks and of oscillatory activity in specific frequency bands.

## 1 Introduction

Adolescence is a crucial stage for physical, psychological, and social development of individuals (Blakemore, 2008; Crone and Dahl, 2012; Dumith et al., n.d.). However, modern teenagers are experiencing unprecedented pressures from academics, social situations, and family dynamics. This has led to a rise in mental health issues, with depression being a significant concern (Huang X. et al., 2023; Farmakopoulou et al., 2024; Muthelo et al., 2024; Samsudin et al., 2024; Urbańska-Grosz et al., 2024). Global research shows that depression is now a major cause of disability in adolescents, with its prevalence increasing over the past decade (Mojtabai et al., 2016). Adolescent depression is marked by persistent feelings of sadness, loss of interest, cognitive difficulties, sleep problems, and challenges in relationships (Whitehouse et al., 2009; Asarnow et al., 2011; Lovato and Gradisar, 2014). In severe cases, it can even result in self-harm and suicidal tendencies. It is therefore imperative to find safe, effective, and easily accessible interventions to address symptoms of adolescent depression.

The adolescent developmental stage, particularly among early high school students aged 15–16, warrants focused investigation (Nelson et al., 2008). This period extends from and builds upon the critical developmental window for prefrontal cortex maturation–a core neural substrate for emotion regulation (Wang et al., 2024) and cognitive control that remains incompletely mature yet retains considerable neuroplasticity into later adolescence (Koechlin et al., 2003; MacDonald et al., 2000). This sustained plasticity provides a continued physiological basis for modulating brain function through interventions such as physical exercise (Thapar et al., 2012). Furthermore, this phase immediately follows the first peak incidence of depressive symptoms, making the study of this age group crucial for understanding the persistence and progression of early-onset vulnerabilities (Desai Boström et al., 2024; Kessler et al., 2001). Consequently, implementing and evaluating interventions within this population holds significant practical importance.

Physical exercise is commonly recommended as a non-pharmacological intervention for depression because of its affordability, minimal side effects, and easy accessibility (El-khalek et al., 2025). Many studies have shown that regular aerobic exercise can enhance mood, decrease anxiety, and benefit physiological functions such as the cardiovascular system (Ruiz et al., 2011). However, traditional exercise regimens may be seen as dull, posing a challenge for individuals with depression who struggle with motivation and enjoyment.

Virtual Reality (VR) technology has emerged as a promising solution to the challenge of engaging users in physical exercise (Jones and Wheat, 2023). By immersing users in interactive 3D virtual environments, VR enhances user engagement and enjoyment (Wagler and Hanus, 2018; Kim and Ko, 2019; Keller et al., 2022). Combining VR with exercise, known as “exergames,” not only maintains the physical benefits of sports training but also makes exercise more appealing and fun through gamification. Research has demonstrated that the audiovisual stimulation provided by VR has been shown to positively impact emotions by diverting attention and enhancing positive emotional experiences (Lavoie et al., 2021; Li et al., 2021; Tao et al., 2024). The application of VR technology in the intervention of depression is a novel and promising auxiliary treatment method (Liu et al., 2025). It provides patients with a safe and engaging intervention experience by creating an immersive and controllable digital environment (Wang et al., 2025a). VR immersive experience modulates large-scale brain network functions through multiple neural mechanisms. The core “sense of presence” originates from the significant suppression of the default mode network (DMN)–when users fully engage their attention in the virtual environment, novel and abundant external sensory inputs continuously occupy cognitive resources, thereby reducing the allocation of resources for internal processes such as self-referential thinking and memory retrieval. This downregulation of DMN activity aligns with neural manifestations observed during high cognitive load tasks (Sridharan et al., 2008), and the depth of immersion is positively correlated with the degree of DMN suppression (Chang et al., 2025). Simultaneously, the continuously changing salient stimuli in the virtual environment enhance the activation of the salience network (SN), enabling it to persistently detect and coordinate the brain’s responses to external events (Hermans et al., 2014). The heightened activity of the SN further facilitates the switching of cognitive resources to the executive control network (ECN), which is consistently activated through goal-directed interactive tasks such as virtual navigation and object manipulation (Menon, 2011; Petersen et al., 2022). Moreover, the VR experience reshapes the dynamic interactions between these networks: the typical antagonistic relationship between the DMN and ECN is reinforced, resulting in stronger anti-correlations (Seeburger et al., 2024), while the SN acts as a dynamic hub, flexibly regulating the brain’s transition from a resting state to a task state in response to real-time fluctuations in event salience and task demands within the virtual environment (An et al., 2024). This synergistic modulation across multiple networks collectively constitutes the neural basis of VR immersion.

Although VR exercise shows promising prospects in improving mental health, its advantages over traditional exercise and the underlying neural mechanisms still need further exploration. Electroencephalogram (EEG) technology provides a high temporal resolution (Coronel-Oliveros et al., 2024; Liu et al., 2024), non-invasive tool (Hodnik et al., 2024; Xie et al., 2024) to objectively assess changes in emotional states (Gong et al., 2024) and explore their neurophysiological basis (Babiloni et al., 2020; Hack et al., 2024). Previous studies have found significant abnormalities in EEG microstates in patients with depression, such as changes in parameters of microstate C related to the default mode network (DMN) and microstate D related to attention networks (Zhao et al., 2022), which are considered as electrophysiological manifestations of neural circuit dysfunction in depression. Additionally, depression is also associated with abnormalities in power spectra of specific frequency bands (e.g., Theta waves) (Bress et al., 2013). However, whether VR exercise can more effectively regulate the brain functional state of depressed adolescents compared to traditional exercise is a scientific question that urgently needs to be answered.

Therefore, this study aims to compare the immediate emotional effects of single-session VR aerobic exercise and traditional aerobic exercise on high school students with depressive symptoms through a randomized crossover controlled trial. Additionally, we will use resting-state EEG technology, specifically microstate and power spectrum analysis, to explore the differences in the effects of these two interventions on brain neural activity, with the goal of uncovering the potential neural mechanisms underlying the improvement of mood through VR exercise.

## 2 Materials and methods

### 2.1 Participants

This study distributed health questionnaires (PHQ-9) (Ettman et al., 2020) to a total of 1608 students in the first and second grades of a high school in Xi’an. The inclusion criteria were: (1) students aged 15–16 years old; (2) right-handed; (3) normal vision or corrected vision. Additional inclusion criteria for the depression group were: PHQ-9 total score ≥ 10 points, confirmed by structured interviews by qualified school counselors, indicating that significant depressive emotional distress exists. The inclusion criteria for the normal control group were: PHQ-9 total score ≤ 4 points. Participants with intermediate PHQ-9 scores (5–9) were excluded from the study. The exclusion criteria were: (1) a history of mental illness (excluding depressive symptoms), neurological disorders, or a family history; (2) taking medications that may affect the central nervous system; (3) the presence of cardiovascular or other physical illnesses unsuitable for moderate-intensity exercise; (4) contraindications related to VR use, such as severe motion sickness.

Using G*Power3.1.9.7 software for sample size estimation, with *f* = 0.25, α = 0.05, Power = 0.90 (Ezegbe et al., 2019), the calculated minimum sample size for a single group is 18 cases. Considering sample loss, ultimately, 40 high school students were involved in this research. The depression group and the healthy control group consisted of 20 individuals each, with 12 females and 8 males in each group. There were no statistically significant differences in age, body mass index (BMI), resting heart rate, and PHQ-9 scores between the two groups (*p* > 0.05) (Table 1). After fully comprehending the research procedures and potential risks. The Ethics Review Committee of Shaanxi Normal University (Approval No. 202516035).

**TABLE 1:** Comparison of baseline characteristics of participants.

Variable	Normal group (*n* = 20)	Depressed group (*n* = 20)	*F*	*P*
Age (years)	15.30 ± 0.17	15.41 ± 0.19	0.20	0.84
BMI (kg/m^2^)	18.97 ± 1.84	18.54 ± 1.67	0.31	0.76
Resting heart rate (bpm)	73.45 ± 1.59	73.13 ± 1.22	1.54	0.22
PHQ-9 score	2.05 ± 1.146	11.90 ± 2.174	6.57	0.014

### 2.2 Experimental procedure

In this study, two groups of participants were required to complete two different types of exercise interventions as follows: (1) “Pure aerobic exercise”: participants performed moderate-intensity (50% ∼ 80% HRmax) cycling exercise (15 min) on a power bike (Sweat Horse JTB616); (2) “VR aerobic exercise”: participants wore a VR head-mounted display (PICO 4, resolution 4320 × 2160, refresh rate 90 Hz) while cycling on the same model of stationary bike. The intervention employed a unified cycling control mode, set in a virtual seaside city environment of the “independent exploration” type, and incorporated the “Ride Bar 2” game program. The VR setup featured the following standardized characteristics: cycling speed was real-time mapped to the forward movement speed in the virtual environment, with the system providing real-time distance prompts and zone-triggered visual feedback (Yin et al., 2016); exercise intensity was strictly controlled within the same range as the pure aerobic exercise condition via heart rate monitoring (Gillen et al., 2012); the visual component presented a seaside landscape under daytime lighting conditions, accompanied by auditory stimuli that included cycling rhythm sounds, waves, and occasional seagull calls to create a multi-channel sensory experience (Moullec et al., 2022). The two protocols were fully consistent in terms of exercise duration, equipment configuration, and intensity control, with the uniqueness of the VR condition lying solely in the enhanced experiential dimension achieved through multi-sensory immersion and gamified interaction.

Participants were randomly and evenly assigned to the two intervention modes, and after a washout period (Mills et al., 2009; Bougrine et al., 2024) (6 h between the two exercise interventions), they underwent the other mode of exercise intervention. EEG data were collected at rest before the first intervention, after “Traditional aerobic exercise,” and after “VR aerobic exercise.” A physical activity monitor (GT3-X+, USA) monitored heart rate changes during exercise. Additionally, after both exercise interventions, participants must fill out the Brief Mood Scale (BFS) questionnaire (Altinsoy and Dikmen, 2025).

### 2.3 Data acquisition and processing

Electroencephalogram data were collected using a 32-channel EEG signal acquisition system from Neuroscan (Brain Vision Recorder, Neuroscan, USA). The electrode cap followed the international 10–20 system for electrode placement (Annaka et al., 2024; Desdentado and Pollatos, 2025). The experiment occurred in a quiet, soundproof room with reduced indoor lighting to minimize distractions. Participants washed their scalp before the experiment, and electrode impedances were adjusted to below 5 kΩ. Participants were instructed to avoid movements such as swallowing, eye movements, frowning, leg shaking, and to maintain a quiet, upright sitting position with their eyes closed and remain awake (Ahsan Awais et al., 2024). Resting-state EEG data were collected before the participant’s first movement and immediately after two subsequent movements, each lasting 6 min (Ng et al., 2024). The EEG signal was sampled at a rate of 1024 Hz, with the mastoid electrodes (M1/M2) used as a reference.

Data processing steps include: (1) importing continuous EEG data; (2) localizing channels; (3) downsampling to 256 Hz; (4) bandpass filtering (0.5–100 Hz) and notch filtering (48–52 Hz) (Alexander et al., 2024); (5) checking waveform plots to identify bad electrodes and interpolating abnormal electrodes; (6) segmenting continuous EEG data into 2-s epochs; (7) using Independent Component Analysis (ICA) to correct data artifacts caused by eye movements, blinks, muscle activity (Electromyogram, EMG), heart activity (Electrocardiogram, ECG), or other non-physiological sources. The selection of independent components was performed manually based on topographic and spectral features; (8) removing electrode data with amplitudes exceeding ±100 μV (Kappenman and Luck, 2010); (9) manually removing segments with large drift; (10) saving the processed EEG data.

### 2.4 EEG microstate analysis

Electroencephalogram microstate analysis is based on the Temporal Atomized Agglomerative Hierarchical Clustering (T-AAHC) algorithm (Chen et al., 2021). (1) Calculate the Global Field Power (GFP), which is the standard deviation of the average potential across all electrodes with a common reference. This is defined as:


$$G\,F\,P\,\sqrt{\frac{\sum_{i=1}^{N}{\left(u\,i-\bar{u}\right)}^{2}}{N}}.$$

Among them, *ui* is the voltage value on the *i* electrode, $\bar{u}$ is the average voltage of all electrodes, and *N* is the number of electrodes in the topography map. GFP is a single quantification index of the topography map for each time point, reflecting the overall intensity of brain electrical activity. Studies have shown that the topographical structure of EEG remains stable when GFP is high, while it changes rapidly near the minimum GFP. Therefore, conducting microstate analysis at GFP peak values can provide the optimal signal-to-noise ratio. In the analysis process, ignoring the polarity of the topography map, each result cluster represents a microstate category. (2) Utilize the microstate analysis software Cartool 3.70 to submit each subject’s EEG topographic map data to the T-AAHC algorithm. This algorithm can identify clusters of topographic maps with similar configurations. The arrangement of microstate categories is determined by the spatial correlation between group-level microstate topographic maps (Chu et al., 2020). (3) Based on the maximum spatial correlation coefficient between each original topographic map and the group-level microstate topographic map, the extracted global microstate topographic maps are “matched” to each subject’s original EEG data stream. The microstate category that matches most at each time point is determined based on spatial correlation (Li et al., 2023). Calculate the following four core microstate parameters (He et al., 2021): (1) Duration: the average duration (ms) of a single microstate appearing continuously. (2) Occurrence: the average number of occurrences of a certain microstate per second. (3) Contribution: the percentage of total time a certain microstate occupies. (4) Transition: the probability of transitioning from one microstate to another.

### 2.5 EEG power spectrum analysis

Electroencephalogram spectral analysis is conducted using MATLAB software for each participant and segment. The analysis method involves using a batch processing approach, with the following steps: For each participant and each segment, Fast Fourier Transform (FFT) is used to obtain the EEG spectrum, transforming the signal from the time domain to the frequency domain, generating a power spectrum (μV^2^) ranging from 1 ∼ 100 Hz. FFT relies on the Discrete Fourier Transform (DFT), where for an N-item complex sequence x(s), its DFT is:


$$X_{\left(K\right)}=\sum_{s=0}^{s-1}x\left(s\right)e^{-j\,\frac{2}{\pi }\,k\,s}\left(K=0,1,2\ldots S-1\right)$$

*X*_(*K*)_ represents the data after DFT transformation, *x*(*s*) is the sampled analog signal, where *x* (*s*) can be imaginary. In reality, *x* (*s*) is all real signals, with the imaginary part being 0. Then the formula can be expanded as:


$$X_{\left(K\right)}=\sum_{s=0}^{s-1}x\left(s\right)\left(\mathrm{cos2}\pi k\frac{n}{N}-\mathrm{jsin2}\pi k\frac{s}{S}\right)\left(K=0,1,2\ldots S-1\right)$$

In the time domain, n represents the s-th sample; x represents the signal time series (*s* = 0,1,2,…,S−1); X stands for the h-th frequency domain representation of the signal x; S represents the signal x corresponding to the k-th frequency component; k corresponds to the k-th frequency component (*k* = 0, 1, 2…, S−1).

Calculate the absolute power of each electrode in different frequency bands (Delta: 1–4 Hz, Theta: 4–8 Hz, Alpha: 8–13 Hz, Beta: 13–30 Hz) in the MATLAB command window. Then, calculate the relative power (percentage of power in a specific frequency band relative to the total power). Increased power in the slow wave δ frequency band during the resting state is associated with enhanced brain plasticity (Castellanos et al., 2010). Frontal θ wave activity may reflect the level of attention (Gevins et al., 1997; Ishii et al., 1999). α waves, as the fundamental rhythm of brain electrical activity, are closely related to the excitatory processes of cortical neurons, alertness (Silber et al., 2007), and working memory (Hsueh et al., 2016). β waves are involved in oscillatory synchronization between motor and somatosensory cortices (Brovelli et al., 2004), and sensorimotor interactions (Tan et al., 2016; Spitzer and Haegens, 2017). Depression symptoms are closely linked to executive and emotional regulation functions. Hence, the experiment focuses on frontal and central regions as regions of interest, and C3, C4, F3, F4, FP1, and FP2 as electrodes of interest.

### 2.6 Statistical analysis

Statistical analyses were performed using SPSS 26.0 software. The data were organized in Excel 2010 and then imported into the statistical software. Normality was assessed using the Shapiro-Wilk test. Data that followed a normal distribution were presented as mean ± standard deviation (M ± SD). A 2 (Group: normal group, depression group) × 3 (Condition: pre-exercise, Post-Traditional Aerobic Exercise, Post-VR-Aerobic Exercise) mixed-design analysis of variance was used to analyze the scores of BFS dimensions and EEG data. The group was the between-subjects variable, while the condition was the within-subjects variable. Simple effects analysis was conducted in case of a significant interaction (*p* < 0.05). For significant main effects, independent samples *t*-tests were used for *post hoc* comparisons. Effect sizes were indicated by partial η^2^ for Analysis of Variance (ANOVA) and Cohen’s d for *t*-tests. The Pearson correlation coefficient was utilized to examine the relationship between changes in the mood scale depression index and changes in EEG microstate temporal parameters and the transition rate index. The significance level for all analyses was set at α = 0.05. The Greenhouse-Geisser correction was applied for variables that did not meet the sphericity assumption. The Bonferroni correction was employed for *post-hoc* tests.

## 3 Results

### 3.1 BFS mood scale results

Results of the two-way mixed design ANOVA (Table 2) revealed that BFS showed significant differences in the dimensions of activity, pleasure, depression, and vigor, with significant main effects of condition, Group, and the interaction between group and condition (*p* < 0.05). Further simple effects analysis revealed: in terms of within-subject effects analysis focusing on condition effects, compared to before exercise, the depression group showed significant increases in both arousal and pleasure ratings after aerobic exercise (*p* < 0.001), while depression and lethargy ratings significantly decreased (*p* < 0.001). Additionally, compared to aerobic exercise alone, the depression group showed significant increases in arousal ratings after VR aerobic exercise and significant decreases in depression and lethargy ratings (*p* < 0.01). There were no significant differences in the normal group (*p* > 0.05). Analysis of inter-subject effects with group as the main factor: the depression group exhibited significantly lower arousal and pleasure scores compared to the normal group at pre-exercise (*p* < 0.001), after aerobic exercise alone (*p* < 0.01), and after VR aerobic exercise (*p* = 0.004, *p* = 0.001). Conversely, the depression group showed significantly higher scores for depression and lethargy than the normal group at pre-exercise (*p* < 0.001), after aerobic exercise alone (*p* < 0.001), and after VR aerobic exercise (*p* < 0.01). No significant differences were observed between the two groups in the remaining components of the scale (*p* > 0.05).

**TABLE 2:** Results of the BFS mood scale.

Scale dimension	Pre-exercise		Post-traditional aerobic exercise		Post-VR-aerobic exercise		*F*-values		
	Normal group	Depressed group	Normal group	Depressed group	Normal group	Depressed group	Condition effect	Group effect	Interaction
Activation	20.350 ± 2.059	8.050 ± 1.986	20.700 ± 2.105	16.650 ± 2.346	20.950 ± 2.212	18.650 ± 2.581	103.304***	144.207***	83.939***
Pleasantness	18.750 ± 2.673	9.350 ± 1.899	19.500 ± 2.039	17.150 ± 2.033	20.450 ± 1.820	18.300 ± 1.922	85.601***	115.186***	45.866***
Contemplation	8.250 ± 1.070	8.050 ± 0.999	8.100 ± 1.021	8.300 ± 1.081	8.000 ± 1.124	8.150 ± 1.040	0.642	0.026	1.927
Calmness	12.800 ± 1.852	12.200 ± 2.191	12.900 ± 1.553	12.400 ± 2.162	13.100 ± 1.373	12.450 ± 2.139	0.247	2.147	0.019
Anger	7.300 ± 1.174	7.900 ± 1.165	7.250 ± 1.164	7.700 ± 1.302	7.050 ± 0.999	7.750 ± 1.293	0.682	3.370	0.264
Excitement	7.750 ± 1.118	7.600 ± 1.142	7.850 ± 1.226	7.700 ± 1.218	7.800 ± 1.240	7.700 ± 1.342	0.144	0.182	0.011
Depression	6.400 ± 1.231	12.050 ± 2.350	6.100 ± 0.968	8.250 ± 1.585	6.000 ± 0.973	6.950 ± 1.050	46.108***	93.624***	33.654***
Lethargy	6.100 ± 1.021	13.100 ± 2.404	6.050 ± 1.099	8.450 ± 1.669	5.700 ± 1.174	7.100 ± 1.119	50.991***	161.117***	41.387***

***p < 0.001.

### 3.2 EEG microstate results

#### 3.2.1 Microstate topographic maps

**FIGURE 1:**
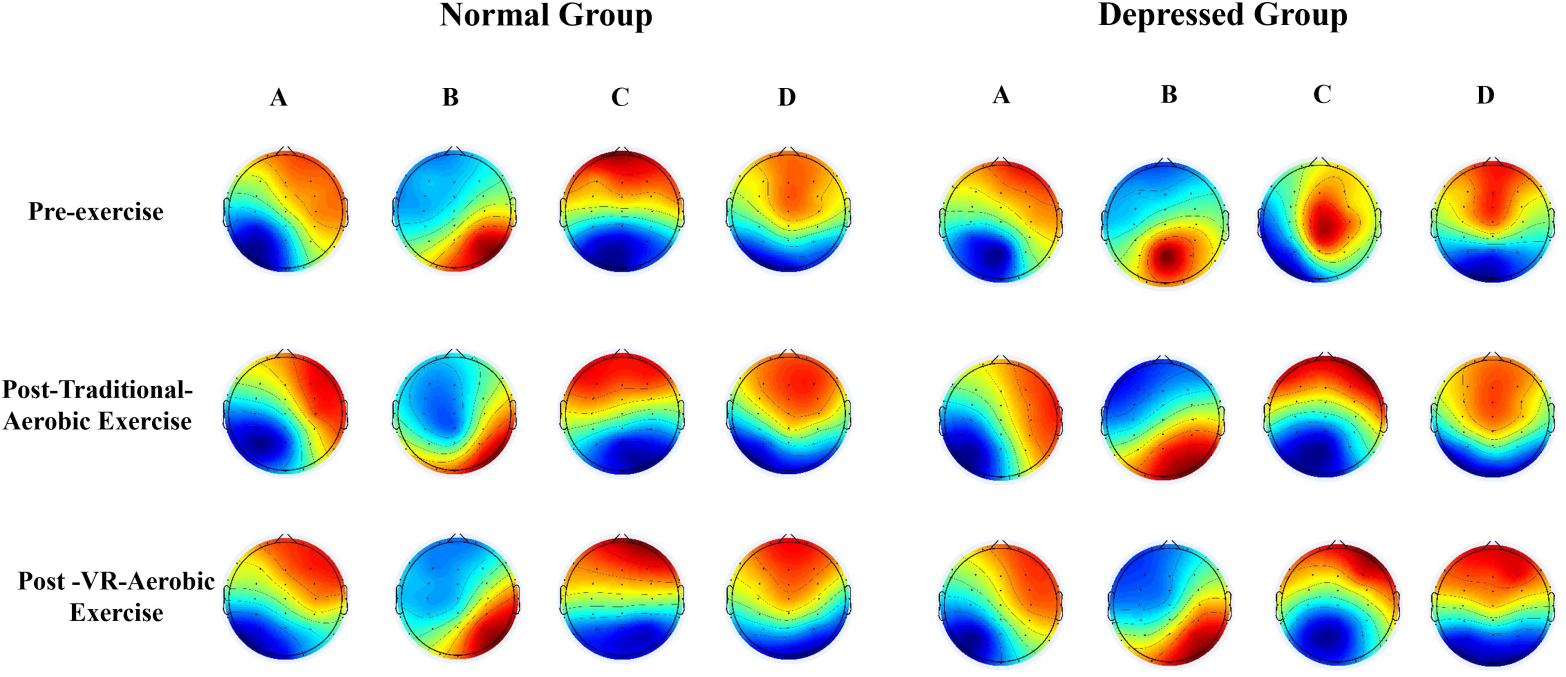
Microstate topographic maps.

As shown in Figure 1, before the exercise intervention, both groups exhibited a similar distribution of centroids in microstates A and D. The centroids of microstate A were located in the right frontal lobe and left occipital lobe, while those of microstate D were in the central frontal lobe and occipital lobe for both groups. However, there were significant differences in the centroid distribution of microstates B and C. In the control group, the centroid of microstate B was in the left frontal lobe and right occipital lobe, while in the depression group, it was in the parietal lobe and left frontal lobe. Similarly, the centroid of microstate C in the control group was in the anterior frontal lobe and the occipital lobe. In contrast, in the depression group, it was in the central region and the left occipital lobe.

After pure aerobic exercise, the centroids of microstates A, B, C, and D in both groups tend to converge toward specific regions in the brain. The centroids of microstate A are located in the right frontal lobe and the left occipital lobe, the centroids of microstate B are located in the left frontal lobe and the right occipital lobe, the centroids of microstate C are located in the frontal lobe and the occipital lobe, and the centroids of microstate D are evenly distributed in the central frontal area and the occipital lobe. Following the VR aerobic exercise intervention, the centroids of microstate topography maps A, B, C, and D in both groups also show similar distributions to those observed after aerobic exercise. This indicates that aerobic exercise intervention can significantly improve the brain’s functional state of depressed high school students, normalizing the distribution of microstate centroids.

#### 3.2.2 Microstate temporal parameters

**FIGURE 2:**
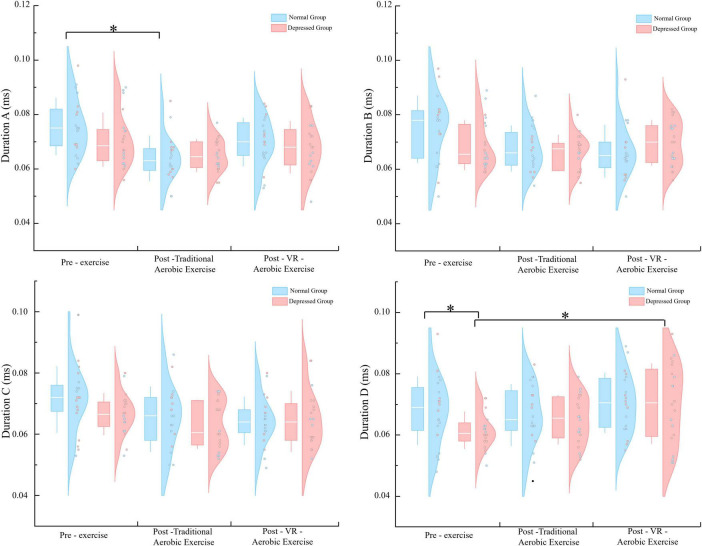
Duration parameters for microstates A–D in the two groups under three. *p < 0.05.

For Duration A, the main effect of condition is significant [*F* (1.699, 64.552) = 8.623, *p* = 0.001, η^2^ = 0.185], with a highly significant difference between pre-exercise and aerobic exercise alone (*p* < 0.01). The condition × group interaction is not significant (*p* = 0.365), but simple effect analysis shows a significant difference for the normal group between pre and post aerobic exercise (*p* < 0.05). For Duration B, neither the main effect of condition nor the condition × group interaction is significant. For Duration C, the main effect of condition is significant [*F* (2, 76) = 3.835, *p* = 0.026, η^2^ = 0.092], with a significant difference between aerobic exercise and pre-exercise (*p* = 0.048). The condition × group interaction is not significant (*p* = 0.495). For Duration D, the main effect of condition is significant [*F* (2, 76) = 4.019, *p* = 0.022, η^2^ = 0.096]. The condition × group interaction is not significant (*p* = 0.317), but simple effect analysis reveals a significant difference between pre-exercise for the normal group and the depression group (*p* = 0.032), and a significant difference between pre - and post - aerobic exercise for the depression group (*p* = 0.047) (Figure 2).

**FIGURE 3:**
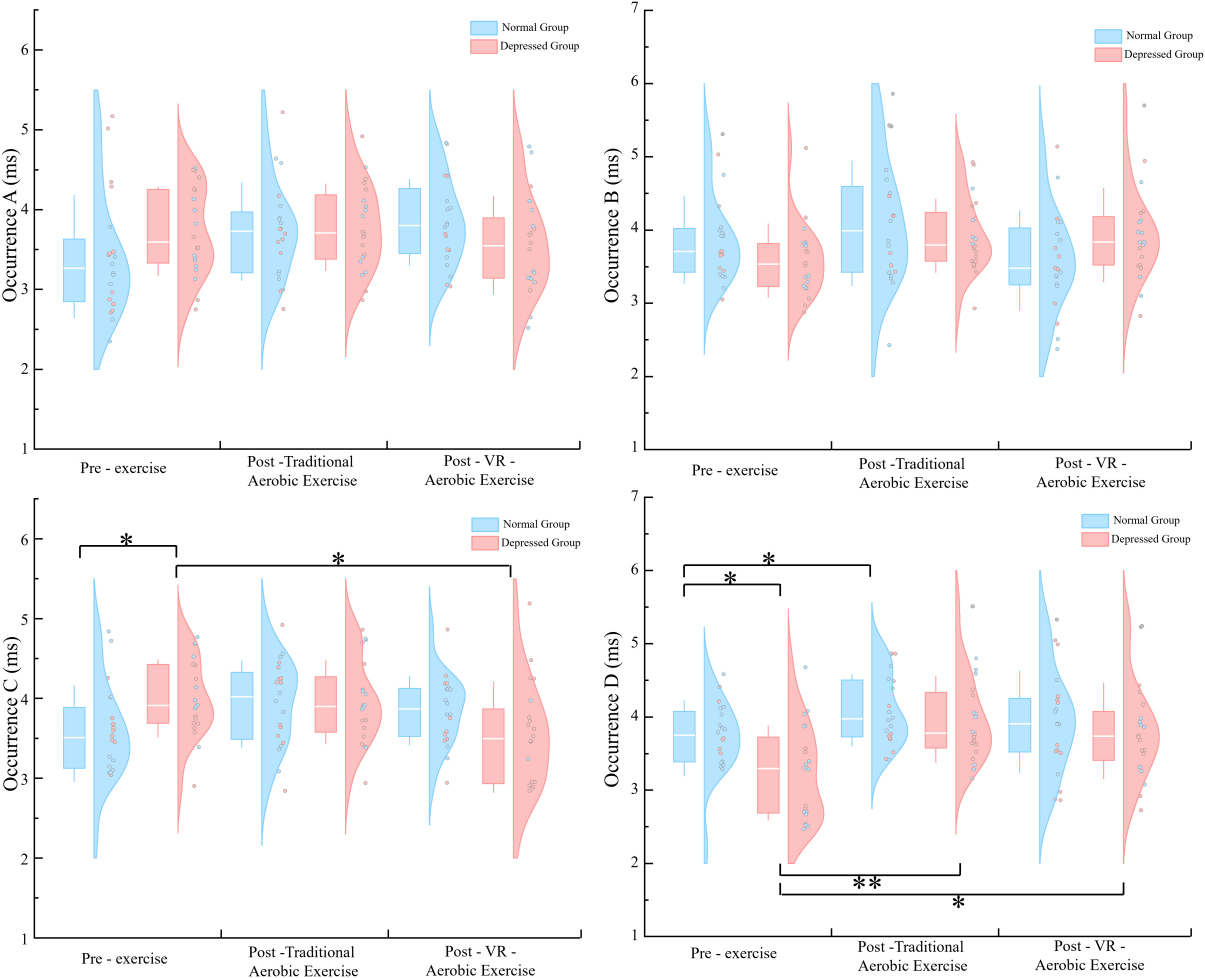
Occurrence parameters for microstates A–D in the two groups under three. *p < 0.05; **p < 0.01.

Occurrence A and Occurrence B: neither the main effect of condition nor the interaction effect of group × condition was significant. Occurrence C: the main effect of condition was not significant [*F* (2, 76) = 2.041, *p* = 0.137, η^2^ = 0.051]; however, there was a significant interaction between group and condition [*F* (2, 76) = 4,430, *p* = 0.015, η^2^ = 0.104]. Simple effect analysis revealed that the depression group scored significantly higher than the normal group before exercise (*p* = 0.016); the depression group showed a significant decrease in VR aerobic exercise post-exercise compared to pre-exercise (*p* = 0.027). Occurrence D: the main effect of condition was statistically significant [*F* (1.629, 61.898) = 10.019, *p* < 0.01, η^2^ = 0.209]. *Post hoc* comparisons indicated a significant improvement in VR aerobic exercise post-exercise compared to pre-exercise (*p* = 0.039); aerobic exercise also showed a significant improvement post-exercise compared to pre-exercise (*p* < 0.01). The interaction between group and condition was not significant (*p* = 0.281). Further simple effect analysis showed that the depression group scored significantly lower than the normal group before exercise (*p* = 0.014); the normal group showed a significant improvement in aerobic exercise post-exercise compared to pre-exercise (*p* = 0.027); the depression group showed a very significant improvement in aerobic exercise post-exercise compared to pre-exercise (*p* < 0.01); and the depression group also showed a significant improvement in VR aerobic exercise post-exercise compared to pre-exercise (*p* = 0.033) (Figure 3).

**FIGURE 4:**
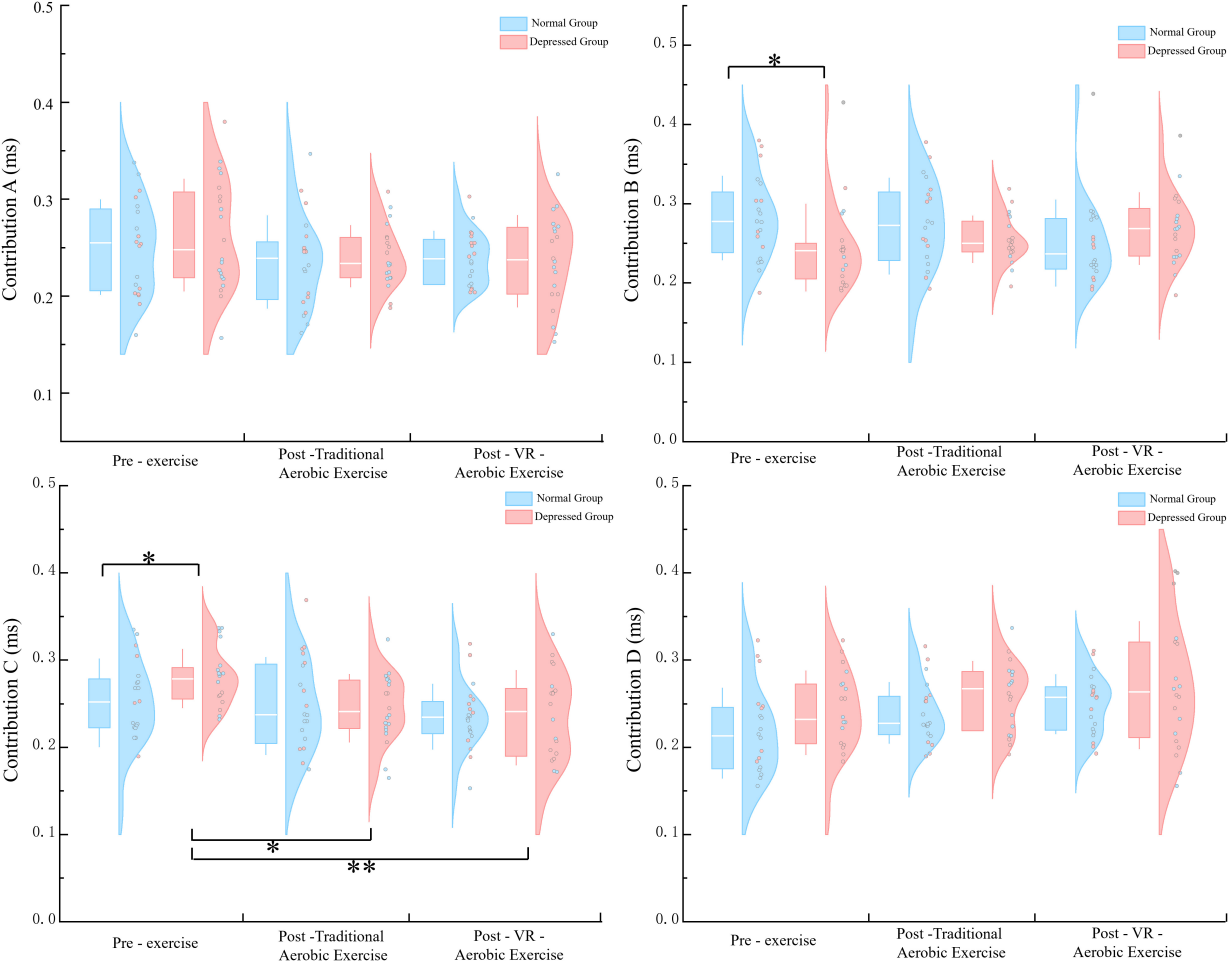
Contribution parameters for microstates A–D in the two groups under three. *p < 0.05; **p < 0.01.

Contribution A: the main effect of condition and the group × condition interaction were both not significant. Contribution B: the main effect of condition and the group × condition interaction were both not significant (*p* = 0.922, *p* = 0.053). Still, simple effect analysis showed a significant difference between the pre-exercise measurements of the depression group and the normal group (*p* = 0.037). Contribution C: the main effect of condition was significant [*F* (2, 76) = 4.917, *p* = 0.010, η^2^ = 0.115], with a significant difference between VR aerobic exercise and pre-exercise measurements (*p* = 0.003). The group × condition interaction was not significant (*p* = 1.542). Still, simple effect analysis revealed a significant difference between the pre-exercise measurements of the depression group and the normal group (*p* = 0.048), as well as significant differences between post-aerobic exercise and post-VR aerobic exercise compared to pre-exercise measurements in the depression group (*p* = 0.042, *p* = 0.002) (Figure 4). Contribution D: the main effect of condition was significant [*F* (1.386, 52.686) = 4.337, *p* = 0.030, η^2^ = 0.102], with a significant difference between aerobic exercise and pre-exercise measurements (*p* = 0.018). The main effect of group was significant [*F* (1, 38) = 6.460, *p* = 0.015, η^2^ = 0.145], with the depression group scoring higher than the normal group (*p* = 0.015). The group × condition interaction was not significant (*p* = 0.955).

#### 3.2.3 Microstate transition probabilities

**FIGURE 5:**
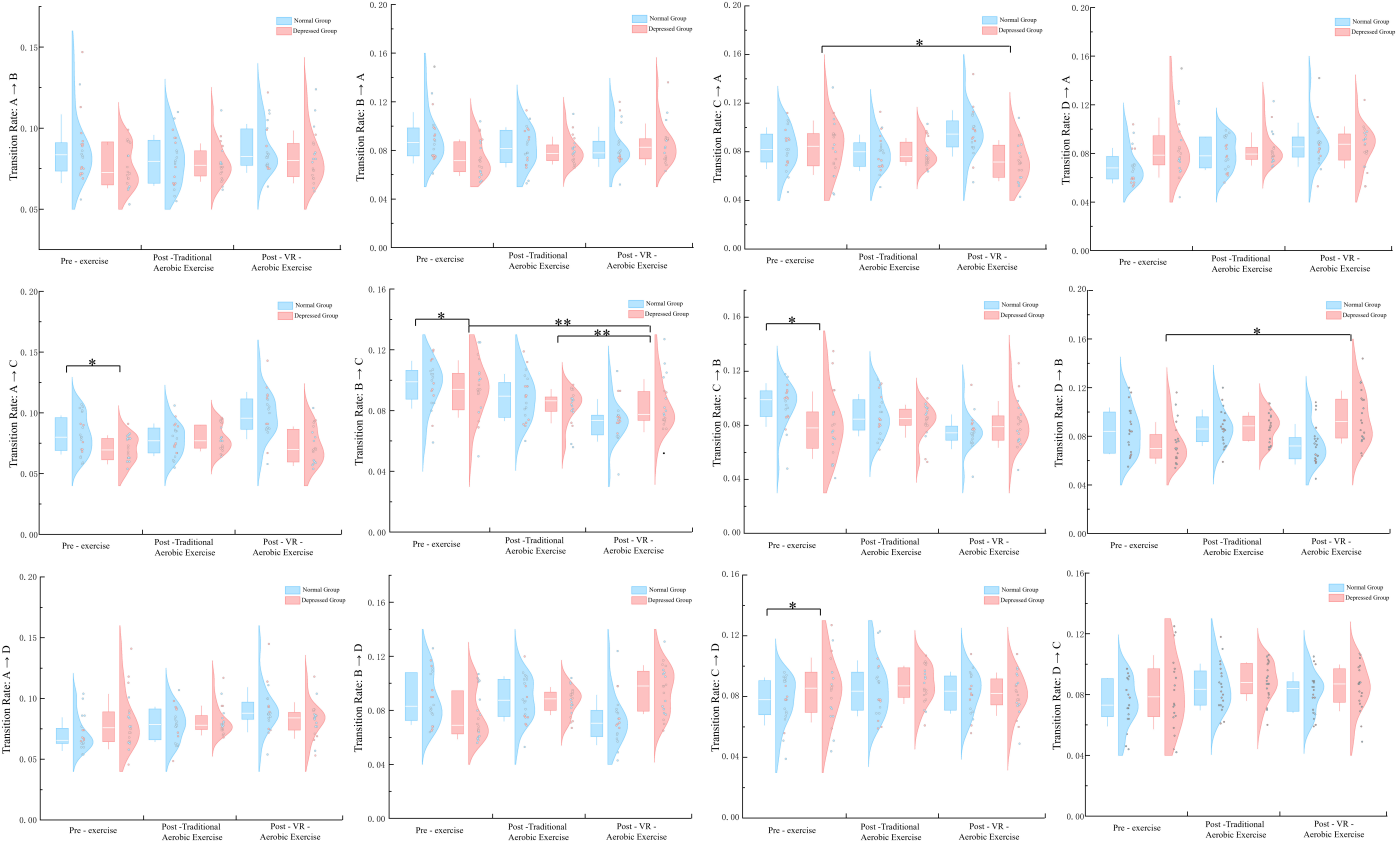
Microstates A–D transition rates: two groups across three conditions. *p < 0.05; **p < 0.01.

According to Figure 5 there are no significant findings for the conversion rates from A → B and A → D: neither the main effect of condition nor the group × condition interaction (*p* > 0.05). As for the conversion rate from A → C, both the main effect of condition and the group × condition interaction are not significant (*p* = 0.612, *p* = 0.135), but further analysis reveals a substantial difference between the depression group and the normal group in pre-exercise (*p* = 0.027).

Conversion rates B → A and B → D: neither the main effect of condition nor the group × condition interaction was significant (*p* > 0.05). Conversion rate B → C: the main effect of condition was significant [*F* (2, 76) = 6.460, *p* = 0.003, η^2^ = 0.145]. Further simple effect analysis revealed that there were significant differences between the VR aerobic exercise post-exercise and pre-exercise, as well as between the VR aerobic exercise post-exercise and the pure aerobic exercise post-exercise (*p* < 0.05); The group × condition interaction was significant [*F* (2, 76) = 4.865, *p* = 0.010, η^2^ = 0.113]. The pre-exercise differences between the depression group and the normal group were significant (*p* = 0.044), and the differences between the post-exercise of VR aerobic exercise and the pre-exercise, as well as between the post-exercise of VR aerobic exercise and the traditional aerobic exercise post-exercise in the depression group were both highly significant (*p* = 0.001, *p* = 0.002).

Conversion rate C → A: the main effect of condition showed significance [*F* (2, 76) = 3.357, *p* = 0.040, η^2^ = 0.081], while the group × condition interaction was not significant (*p* = 0.367). Further analysis of simple effects revealed a substantial difference in VR aerobic exercise post-exercise compared to pre-exercise for the depression group (*p* < 0.05). Conversion rates C → B, C → D: neither the main effect of condition nor the group × condition interaction was significant (*p* > 0.05). However, simple effect analysis showed significant differences between the depression and normal Groups in pre-exercise measurements for these two conversion rates (*p* < 0.05).

Conversion rates from D → A and from D → C: the main effect of condition is not significant, and the group × condition interaction is also not significant (*p* > 0.05). Conversion rate from D → B: the main effect of condition is substantial [*F* (2, 76) = 4.082, *p* = 0.021, η^2^ = 0.097]. *Post hoc* comparisons showed a significant difference in VR aerobic exercise compared to the pre-exercise (*p* < 0.05). The group × condition interaction is not essential, but simple effect analysis revealed a significant difference in the depression group after VR aerobic exercise compared to the pre-exercise (*p* < 0.05).

#### 3.2.4 Correlations between microstates and mood scale scores

In comparison with the pre-exercise assessment, significant changes were observed in the post-exercise BFS mood scale in terms of liveliness, pleasantness, depression, and lack of vitality. After aerobic exercise alone, Occurrence D and Contribution C showed significant changes. Following VR aerobic exercise, Duration D, Occurrence C, Occurrence D, Contribution C, transition rate B → C, and transition rate D → B all exhibited significant changes.

**FIGURE 6:**
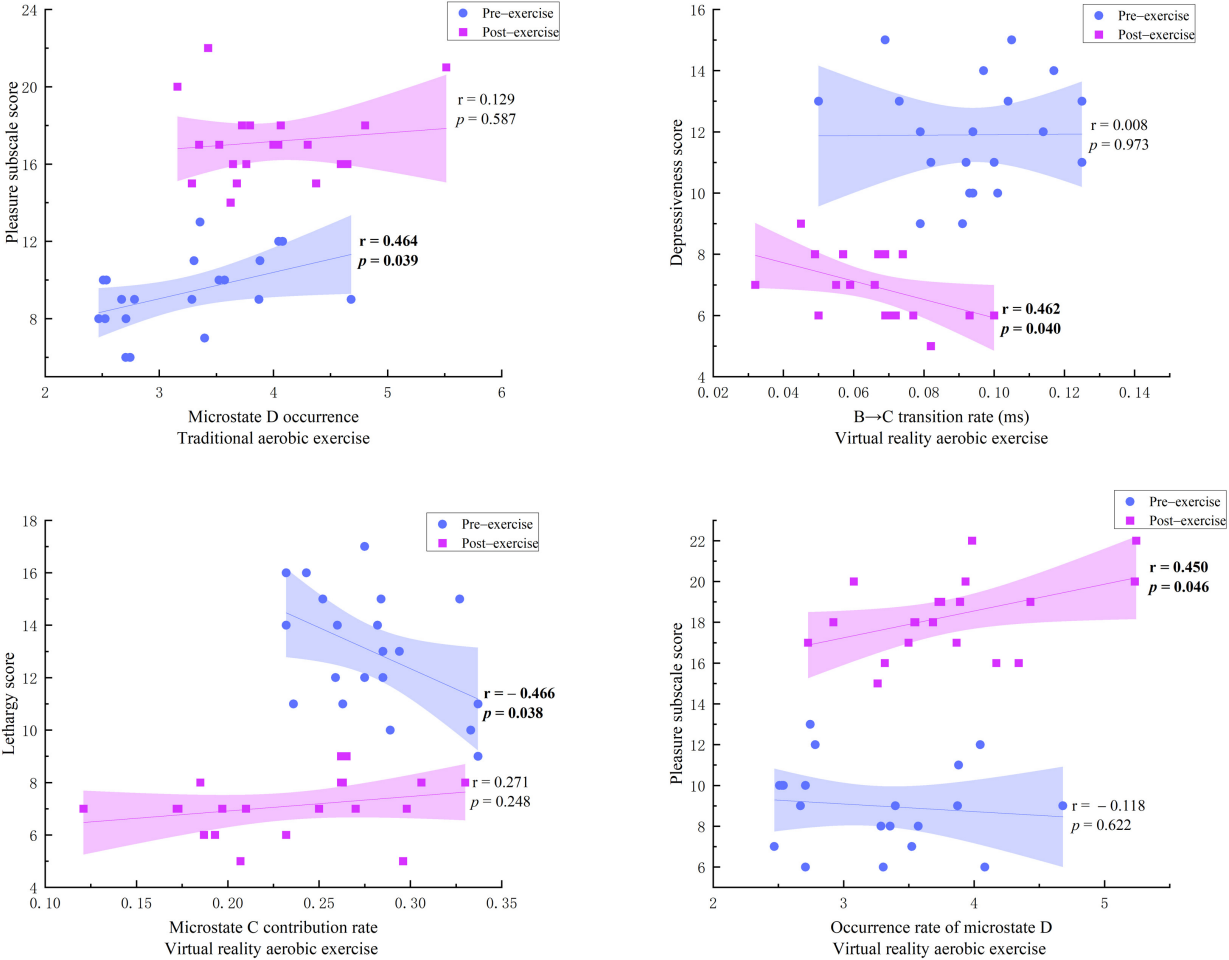
Correlations between mood scale scores and EEG microstate changes.

During pure aerobic exercise, as shown in Figure 6, there was a moderate positive correlation between pre-exercise pleasure and Occurrence D (*r* = 0.464, *p* = 0.039), but this correlation disappeared after exercise (*r* = 0.129, *p* = 0.587). This suggests that pure aerobic exercise may weaken the linear relationship between the two by restructuring the brain’s functional network, supporting the “network flexibility hypothesis” of exercise improving mood – that mood enhancement may stem from optimizing brain information processing patterns from static network activation to dynamic network interaction, rather than changes in a single microstate parameter. The other BFS mood component scale indicators and microstate time parameters showed no statistical significance.

Before the VR aerobic exercise intervention, there was no significant correlation between the B → C transition rate and depression (*r* = 0.008, *p* = 0.973), indicating no clear relationship between the severity of depressive symptoms at rest and the dynamic switching ability of the brain microstates. However, after the VR aerobic exercise intervention, there was a significant moderate negative correlation between them (*r* = −0.462, *p* = 0.04), suggesting that individuals with higher levels of depression had lower B → C transition rates after exercise, indicating that VR aerobic exercise may improve emotional regulation in individuals with depression by enhancing the flexibility of brain functional networks. Before the exercise, there was a significant moderate negative correlation between the level of inactivity and Contribution C (*r* = −0.466, *p* = 0.038), indicating that individuals with more severe symptoms of inactivity had lower Contribution C rates at rest. However, this correlation disappeared after the exercise (*r* = 0.271, *p* = 0.248), suggesting that VR aerobic exercise weakened the linear relationship between inactivity and Contribution C, improving individuals’ energy levels and subjective vitality. Before the exercise, there was no significant correlation between pleasure and Occurrence D (*r* = −0.118, *p* = 0.622). However, after the exercise, there was a significant moderate positive correlation between them (*r* = 0.450, *p* = 0.046), indicating that individuals with higher levels of pleasure had higher occurrence rates of Occurrence D after the exercise.

### 3.3 EEG relative power results

Theta frequency band: the main effect of the condition was highly significant for channels C3, C4, F3, F4, FP1, and FP2 (*p* < 0.01). *Post hoc* comparisons showed that the EEG values after pure aerobic and VR aerobic exercise were significantly lower than pre-exercise values (*p* < 0.01) for all channels. There was no significant interaction between group and condition for any channel. Regarding group differences, the depression group had significantly higher EEG values than the normal group on channels F4 (*p* = 0.004) and FP1 (*p* = 0.013). Simple effect analysis revealed that the depression group had significantly higher pre-exercise EEG values than the normal group on channels C3, C4, F3, F4, and FP1 (*p* < 0.05). The normal and depression groups showed significantly lower EEG values after pure aerobic exercise and VR aerobic exercise compared to their respective pre-exercise values (*p* < 0.01) on these channels. On channel FP2, both groups had significantly lower EEG values after pure aerobic exercise and VR aerobic exercise compared to their pre-exercise values (*p* < 0.01), with no significant difference between the groups in the pre-exercise values (*p* > 0.05) (Table 3).

**TABLE 3:** Relative power values of normal and depressed groups across three states.

Frequency band	Channel	Pre-exercise		Post-traditional aerobic exercise		Post-VR-aerobic exercise	
		Normal group	Depressed group	Normal group	Depressed group	Normal group	Depressed group
Theta	C3	0.221 ± 0.007	0.225 ± 0.005*	0.209 ± 0.005**	0.208 ± 0.006^##^	0.210 ± 0.006**	0.210 ± 0.006^##^
	C4	0.222 ± 0.008	0.225 ± 0.006	0.208 ± 0.004**	0.208 ± 0.006^##^	0.209 ± 0.005**	0.210 ± 0.006^##^
	F3	0.222 ± 0.009	0.224 ± 0.005	0.207 ± 0.005**	0.208 ± 0.005^##^	0.208 ± 0.004**	0.207 ± 0.006^##^
	F4	0.223 ± 0.009	0.229 ± 0.006*	0.208 ± 0.005**	0.209 ± 0.005^##^	0.206 ± 0.004**	0.209 ± 0.005^##^
	FP1	0.218 ± 0.006	0.223 ± 0.007*	0.207 ± 0.005**	0.209 ± 0.004^##^	0.206 ± 0.006**	0.208 ± 0.005^##^
	FP2	0.222 ± 0.009	0.222 ± 0.006	0.207 ± 0.004**	0.208 ± 0.004^##^	0.209 ± 0.006**	0.211 ± 0.006^##^
Alpha	C3	0.235 ± 0.011	0.238 ± 0.008	0.218 ± 0.011**	0.217 ± 0.012^##^	0.219 ± 0.007**	0.221 ± 0.006^##^
	C4	0.238 ± 0.014	0.239 ± 0.008	0.215 ± 0.013**	0.216 ± 0.010^##^	0.216 ± 0.006**	0.218 ± 0.007^##^
	F3	0.240 ± 0.013	0.242 ± 0.011	0.217 ± 0.012**	0.218 ± 0.011^##^	0.218 ± 0.006**	0.220 ± 0.008^##^
	F4	0.239 ± 0.014	0.242 ± 0.012	0.215 ± 0.012**	0.218 ± 0.010^##^	0.216 ± 0.006**	0.219 ± 0.006^##^
	FP1	0.236 ± 0.011	0.241 ± 0.010	0.216 ± 0.011**	0.220 ± 0.011^##^	0.215 ± 0.011**	0.220 ± 0.007^##^
	FP2	0.237 ± 0.010	0.241 ± 0.011	0.215 ± 0.011**	0.218 ± 0.010^##^	0.217 ± 0.006**	0.220 ± 0.005^##^
Beta	C3	0.192 ± 0.006	0.190 ± 0.010	0.182 ± 0.003**	0.182 ± 0.005^##^	0.184 ± 0.004**	0.184 ± 0.005^#^
	C4	0.191 ± 0.006	0.192 ± 0.008	0.182 ± 0.002**	0.183 ± 0.006^##^	0.183 ± 0.004**	0.184 ± 0.005^##^
	F3	0.193 ± 0.012	0.191 ± 0.009	0.183 ± 0.005**	0.181 ± 0.005^##^	0.186 ± 0.005*	0.184 ± 0.005^#^
	F4	0.190 ± 0.010	0.188 ± 0.011	0.182 ± 0.005**	0.181 ± 0.004^#^	0.182 ± 0.004**	0.181 ± 0.004^#^
	FP1	0.194 ± 0.008	0.190 ± 0.006	0.183 ± 0.005**	0.180 ± 0.004^##&^	0.184 ± 0.004**	0.181 ± 0.005^##°^
	FP2	0.190 ± 0.007	0.190 ± 0.007	0.182 ± 0.005**	0.181 ± 0.004^##^	0.185 ± 0.005*	0.183 ± 0.005^##^

Compared with the normal group pre-exercise, *p < 0.05; **p < 0.01; compared with the depressed group pre-exercise, ^#^*p* < 0.05; ^##^*p* < 0.01; compared with the normal group post-traditional aerobic exercise, ^&^*p* < 0.05; compared with the normal group post-VR-aerobic exercise, °*p* < 0.05.

Alpha frequency band: the main effects of C3, C4, F3, F4, FP1, and FP2 channels on the condition are all highly significant (*p* < 0.01). The EEG values after pure aerobic exercise and VR aerobic exercise are both significantly lower than the pre-exercise (*p* < 0.01). The group effect is significant for FP1 and FP2 channels (*p* = 0.022, *p* = 0.038), with the depression group showing significantly higher EEG values than the normal group (*p* < 0.05). There are no significant group × condition interactions for all channels. Simple effect analysis shows that in C3, C4, F3, F4, FP1, and FP2 channels, the EEG values after pure aerobic exercise and VR aerobic exercise for both normal and depression groups are significantly lower than their respective pre-exercise (*p* < 0.01), and both groups show consistent trends in changes in each condition.

The beta frequency band showed highly significant main effects on the state for C3, C4, F3, F4, FP1, and FP2 channels (*p* < 0.01). The EEG values after traditional aerobic exercise and VR aerobic exercise were both significantly lower than those before the exercise (*p* < 0.01). The main effect of group was highly significant for the FP1 channel (*p* = 0.001), with the EEG values of the depression group significantly lower than those of the normal group (*p* < 0.01). However, the main effect of group was not significant for the other channels. There were no significant interactions between group and state for any of the channels. Simple effect analysis showed that in the C3, C4, F3, F4, and FP2 channels, the EEG values of both the normal and depression groups after simple/VR aerobic exercise were significantly lower than those before the exercise (*p* ≤ 0.025). In the FP1 channel, the EEG values of the depression group after simple and VR aerobic exercise were not only significantly lower than their respective pre-exercise values (*p* < 0.01), but also significantly lower than the corresponding post-exercise values of the normal group (*p* ≤ 0.048).

## 4 Discussion

The study investigated the effects of a single session of VR aerobic exercise on middle school students with depressive symptoms. The results showed that VR aerobic exercise was more effective than traditional aerobic exercise in improving the emotional state of students with depression, particularly in increasing activity and reducing depression. EEG analysis revealed that VR aerobic exercise could normalize abnormal resting-state brain network dynamics in depressed adolescents and modulate the brain’s electrical power spectrum related to emotions and arousal.

### 4.1 Analysis of mood scale results

A single session of moderate-intensity aerobic exercise can effectively improve negative emotions and enhance positive emotions in adolescents with depression, as shown in this study. This aligns with the many past studies on the antidepressant benefits of exercise (Jacka et al., 2017; Firth et al., 2019; Thapa et al., 2023). Students in the depression group had higher activity scores after completing VR aerobic exercise compared to traditional aerobic exercise, with lower scores of depression and lethargy. Combining immersive virtual reality with exercise can significantly improve the emotional state of the depression group compared to exercise alone.

Virtual reality’s immersive and engaging experience may contribute to its superiority over traditional exercise environments (Bird et al., 2021). The virtual world created by VR can distract users from negative emotions and physical discomfort, enhancing pleasure during exercise (Ekkekakis, 2003). Gamified forms of exercise in VR can increase fun and motivation, which is beneficial for individuals with motivation deficits or symptoms of depression (Muñoz et al., 2022). The dynamic visual and auditory stimuli in VR environments provide richer sensory inputs, potentially positively regulating brain function and emotions (Tao et al., 2024). A recent study with college students found that an 8-weeks VR exercise intervention was more effective than traditional aerobic exercise in improving mood disturbances, tension, anger, depression, and vitality (Wang et al., 2025b). This evidence suggests that VR can amplify the psychological benefits of exercise.

### 4.2 Analysis of microstate findings

The research findings revealed that the depressed group displayed notable abnormalities in the distribution of microstate B and C centroids at baseline, supporting the theory of disrupted brain network functional connectivity in depression (Zhang et al., 2011; Guo et al., 2025). Following the exercise intervention, the centroid distribution of the four microstates in both groups appeared to normalize, suggesting that exercise plays a crucial role in regulating brain function states.

The study found that after VR exercise, the occurrence rate of microstate C decreased significantly in the depression group. Microstate C is linked to the salience network (Nishida et al., 2025), which includes the anterior cingulate cortex and insula (Menon and Uddin, 2010), important for emotion regulation (Fang et al., 2024; Roberts and Mulvihill, 2024). The decrease in the occurrence of Microstate C reflects how the VR experience redistributes attentional resources, reducing the excessive monitoring of internal negative states–a process potentially mediated by the normalization of anterior cingulate cortex and insula activity–thereby helping to alleviate rumination in individuals with depression. Additionally, the occurrence rate and contribution of microstate D increased after exercise, indicating improved function of the dorsal attention network. This improvement may be due to the sensory stimulation and task demands in the VR environment, which help shift individuals from internal contemplation to external task engagement. The depression group showed a significant increase in B → C transition rate after VR exercise, which was negatively correlated with the improvement of depressive symptoms. This suggests enhanced efficiency of information transmission between visual networks and salience networks. The increased flexibility in network collaboration reflected by the increase in transition rate may be a key mechanism in breaking the characteristic cognitive rigidity of depression. The occurrence rate of microstate D after VR exercise was positively correlated with feelings of pleasure (Bai et al., 2024), while the B → C transition rate was negatively correlated with the severity of depression (Luo et al., 2025). This links specific changes in network dynamics directly to emotional improvement (Weiss et al., 2024). In contrast, single aerobic exercise could also change some microstate parameters, but did not show similar systematic neural-behavioral correlations, highlighting the unique advantage of VR intervention in regulating brain network dynamics.

These findings collectively support the applicability of the “network flexibility hypothesis” in explaining the mechanism of how exercise improves mood. VR exercise not only regulates the activity levels of specific brain networks but also promotes flexible transitions of brain functional states by enhancing dynamic interactions between networks. The synergistic effect of normalizing network activity and enhancing attention network function drives individuals to shift from internal reflective modes to external engaged modes, providing a new theoretical perspective for understanding the neural mechanisms of how VR-enhanced exercise improves depressive mood.

### 4.3 Analysis of power spectrum results

The depression group showed significantly higher Theta relative power in the F4 and FP1 channels in the frontal lobe, and higher Alpha relative power in the FP1 and FP2 channels in the anterior frontal lobe compared to the control group. The increase in the Theta band may be related to emotional regulation dysfunction and weakened cognitive control in depression. While some studies suggest a decrease in theta activity among individuals with depression (Zandbagleh et al., 2024), other research has indicated an increase in theta power (Huang Y. et al., 2023). This discrepancy could be attributed to sample heterogeneity or variations in analysis techniques. The higher Alpha power in the anterior frontal lobe in the depression group may reflect an enhanced inhibitory or deactivating state in that region, consistent with reduced executive function and motivation in depression patients (Gotlib, 1998; Huang Y. et al., 2023). In addition, the depression group had significantly lower Beta power in the left frontal lobe (FP1 channel) compared to the healthy group, which may indicate an insufficient cortical arousal level or decreased cognitive processing ability, consistent with symptoms of cognitive slowing and lack of concentration in depression.

Both exercise interventions significantly decreased relative power in the Theta, Alpha, and Beta frequency bands for all participants. A decrease in low-frequency power is a common finding after acute aerobic exercise and is generally interpreted as an indicator of increased cortical arousal and activation. The baseline differences we observed, such as higher theta and alpha power in the depressed group, are also consistent with literature suggesting altered cortical arousal and information processing in depression. However, we found no significant additional effect of VR on these power spectrum measures. This negative finding is critical because it indicates that the superior mood-enhancing properties of VR are not simply due to a greater change in overall cortical arousal. Instead, the advantage of VR appears to lie in its ability to more specifically reorganize the temporal dynamics of large-scale brain networks, a phenomenon captured by microstate analysis but overlooked by traditional spectral analysis. This highlights the methodological value of employing microstate analysis to uncover subtle yet functionally significant changes in brain activity.

### 4.4 Study limitations and future directions

Although this study systematically explored the immediate effects of VR-enhanced aerobic exercise on mood and brain neural activity in depressed adolescents and yielded some positive findings, several limitations exist, which also point the way for future research.

(a) This study is an acute-effect investigation of a single bout of exercise intervention; thus, its results cannot address the long-term benefits of VR exercise. The satisfaction derived from VR might be transient, and the short-term design of this study cannot verify the persistence of its effects, nor can it assess whether long-term use might lead to dependency or potential negative mood impacts due to increased screen time. Future research needs to test the cumulative effects and long-term stability of VR exercise through trials involving multiple interventions and long-term follow-ups. (b) The intervention protocol in this study (e.g., VR scenario, exercise intensity, and duration) was standardized. While this design ensured internal validity, it did not account for individual differences in depressive symptom profiles, exercise preferences, and baseline neural activity levels, thus failing to achieve truly personalized intervention. Future research could explore adaptive intervention protocols based on individual FMS or EEG baseline characteristics. (c) Caution is warranted in interpreting the neural mechanisms. The observed changes in EEG microstates and power spectra were associated with mood improvement, but this correlation does not equate to causation. It remains difficult to fully disentangle to what extent these changes in neural indicators are due to the physiological effects of exercise itself versus the psychological effects induced by VR immersion. Future studies could incorporate neuroimaging techniques with higher spatial resolution, such as fMRI, to more precisely localize activity changes in relevant brain networks. (d) Regarding the washout period implemented in this study, although the 6-h interval was determined based on physiological recovery time and precedents in relevant research–and substantially exceeds the time required for the normalization of key physiological parameters–we acknowledge that residual effects on subjective psychological states such as mood may persist beyond this duration. This represents a limitation of the present study. Future research employing longer washout periods or between-subjects designs would help to clarify this issue. (e) The spectral analysis of this study focused on comparing the relative power of ICA-derived source components (reflecting large-scale brain networks) across groups and conditions. Therefore, we did not perform cluster-based or permutation-based statistical mapping of scalp topography. This approach was chosen to prioritize physiologically interpretable source-level signals and to account for the limited spatial resolution and volume conduction effects inherent in 32-channel EEG data. Future studies may employ high-density EEG or combined EEG-fMRI to better resolve the spatial topography and neural origins of these spectral changes.

## 5 Conclusion

This study demonstrates that a single session of VR-enhanced aerobic exercise is superior to traditional aerobic exercise in immediately improving mood states in adolescents with depressive symptoms. While both interventions enhanced activation and pleasure while reducing depression and lethargy, VR elicited significantly greater emotional benefits. At the neurophysiological level, VR exercise specifically modulated key microstate parameters–reducing the occurrence of salience-network-related microstate C, prolonging attention-network-related microstate D duration, and enhancing transitions between visual-salience networks. These changes correlated with mood improvement and suggest enhanced dynamic coupling between large-scale brain networks. In contrast, spectral power changes indicated general cortical arousal without modality-specific differences. The findings support VR-enhanced exercise as an effective, engaging intervention for acutely alleviating depressive mood in adolescents. Future studies should investigate its long-term efficacy, optimal dosing parameters, and personalized application through longitudinal designs and mechanistic exploration.

## Data Availability

The original contributions presented in this study are included in this article/supplementary material, further inquiries can be directed to the corresponding author.
